# Supplementary figures and images for: A comparison of lodgepole and spruce needle chemistry impacts on terrestrial biogeochemical processes during isolated decomposition
Source: PeerJ. 2020 Jul 16;8:e9538. doi: 10.7717/peerj.9538 (PMC7369028; doi:10.7717/peerj.9538)

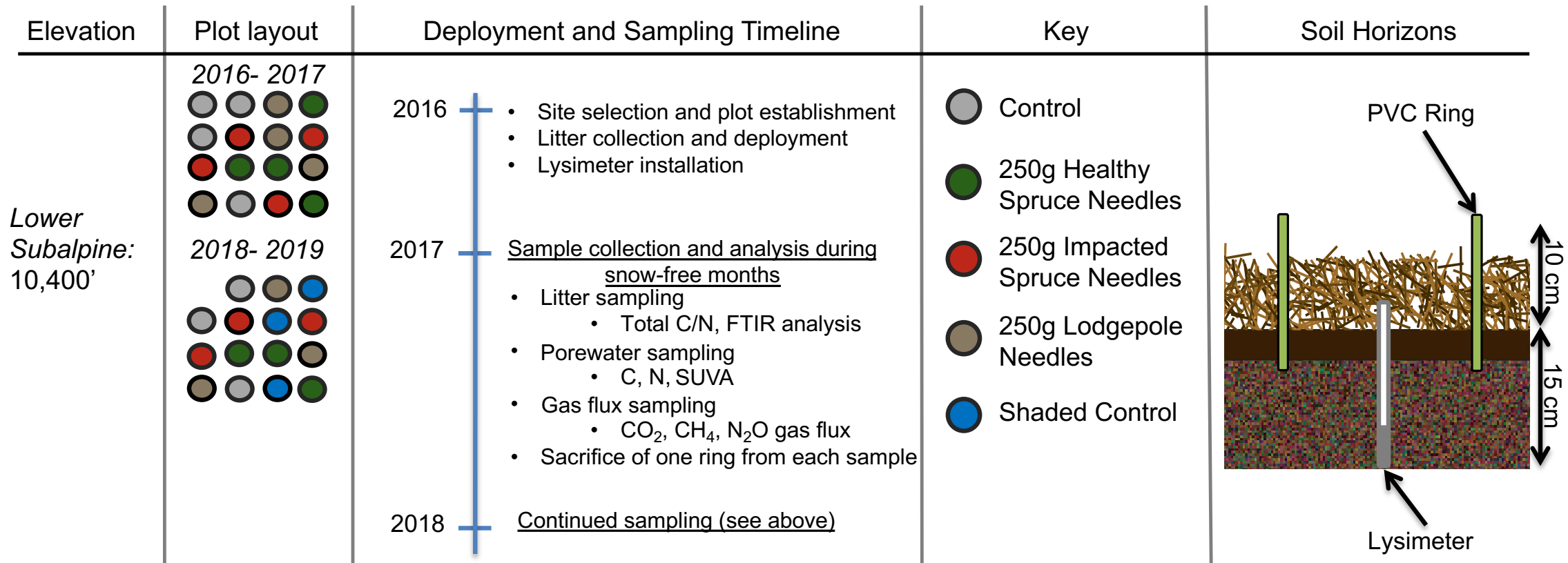

Supplement: Supplemental Information 1 — Randomized placement of each sample collar is shown in the Plot Layout. In 2016-2017 there were quadruplicate collars for each sample type. This changed at the end of 2017 when one of each sample quadruplicate was sacrificed. This led to triplicates of each collar in 2018 and the addition of shade controls. [file peerj-08-9538-s001.pdf]

2017

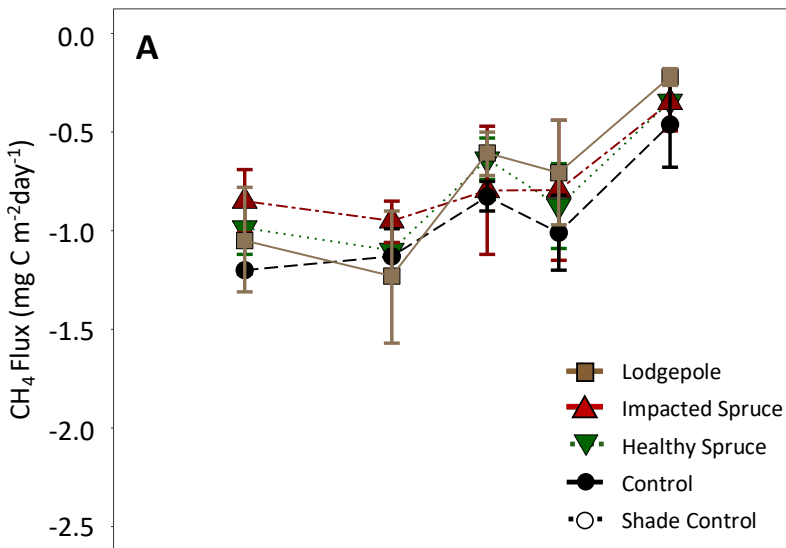

2018

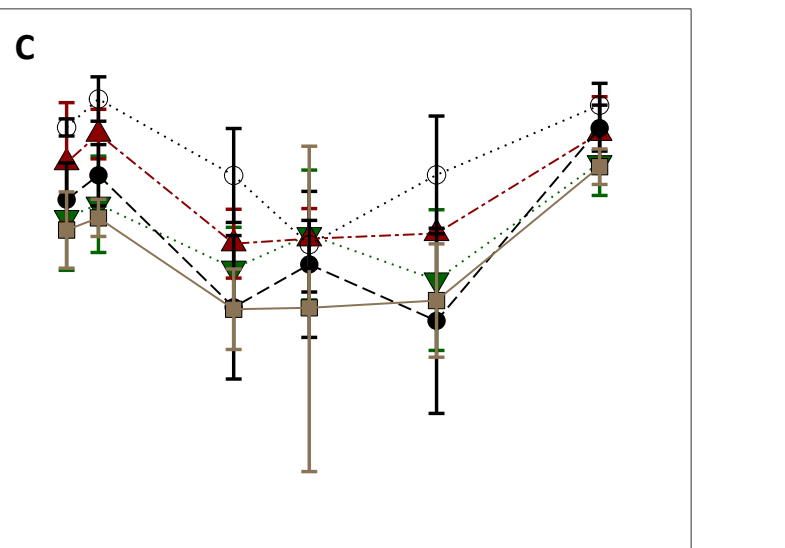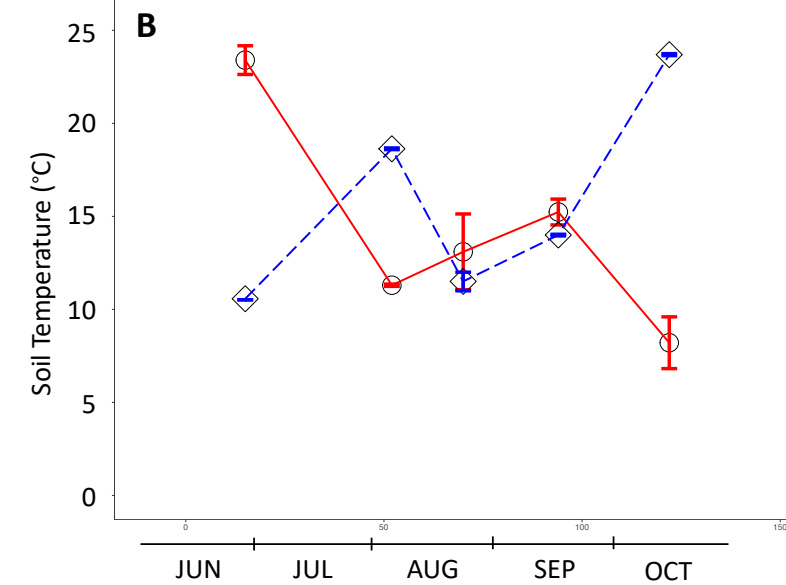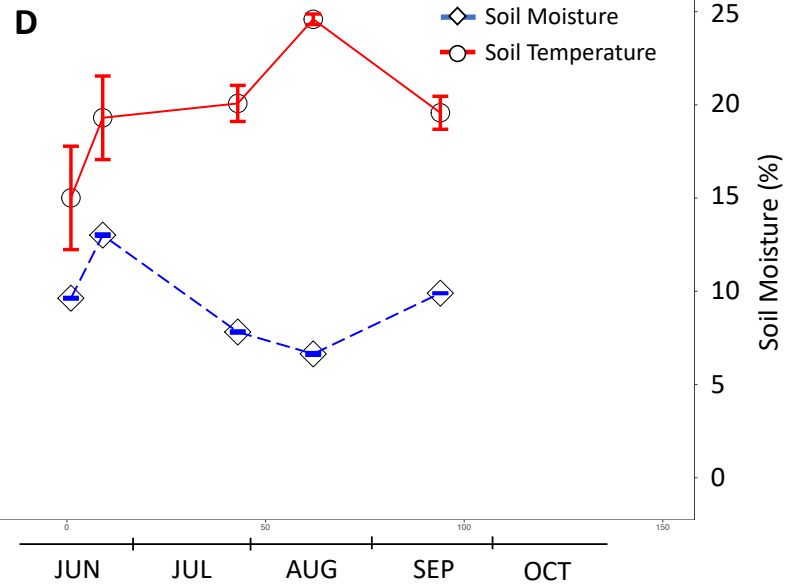

Supplement: Supplemental Information 2 — (A,C) Measured methane above decomposing needle collars and (B,D) seasonal variables of local soil at the experimental plot over the summer months of 2017 (A,B) and 2018 (C,D). Error bars indicate plus or minus one standard deviation (n = 3 or 4). The fifth point in September 2018 for impacted spruce is missing a third measurement, as result no error bars are present. [file peerj-08-9538-s002.pdf]

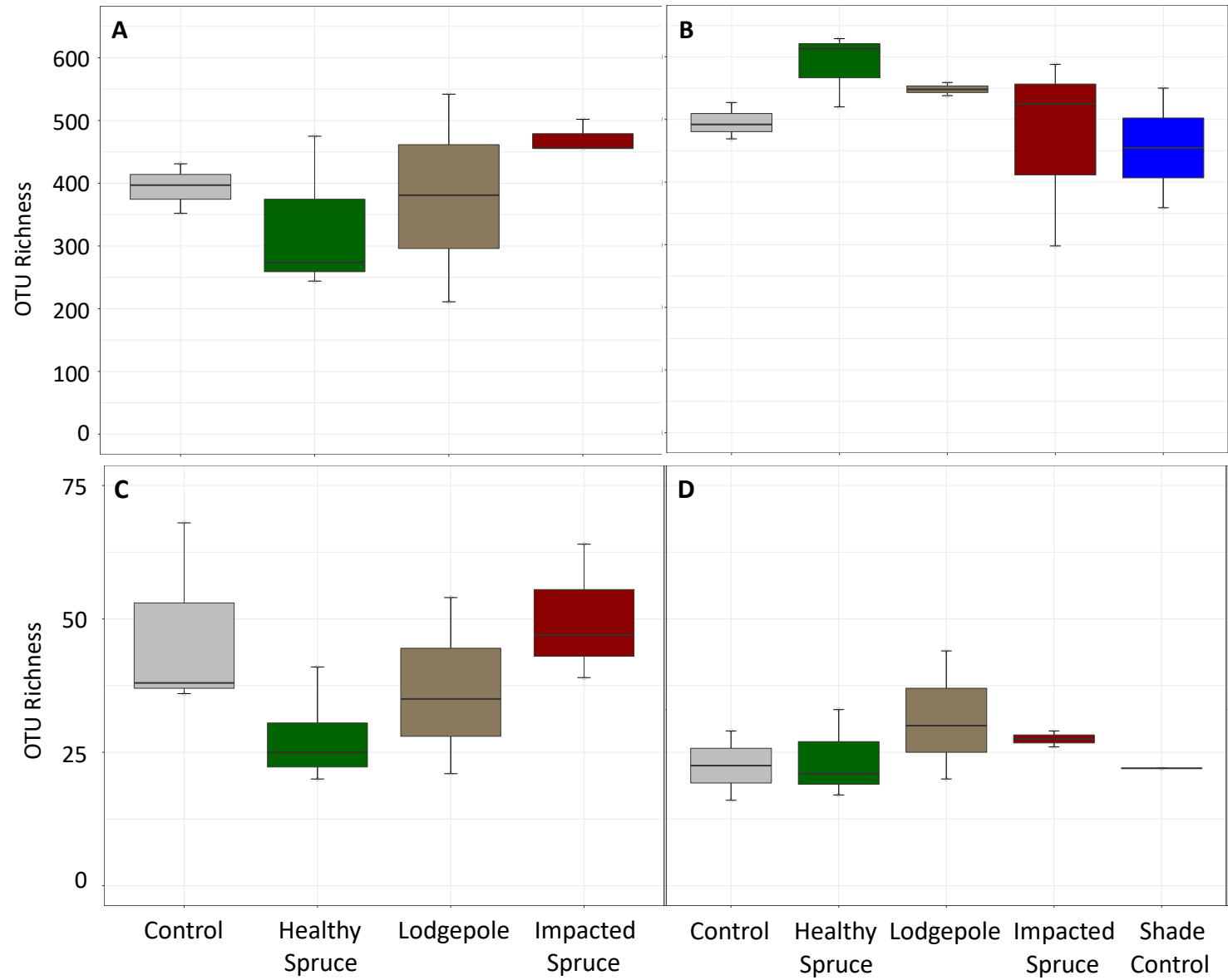

Supplement: Supplemental Information 3 — (A,B) bacterial, and (C,D) fungal communities in the Lower Subalpine plot for dates (A,C) August 2017 and (B,D) July 2018. [file peerj-08-9538-s003.pdf]

# Phylum; Genus

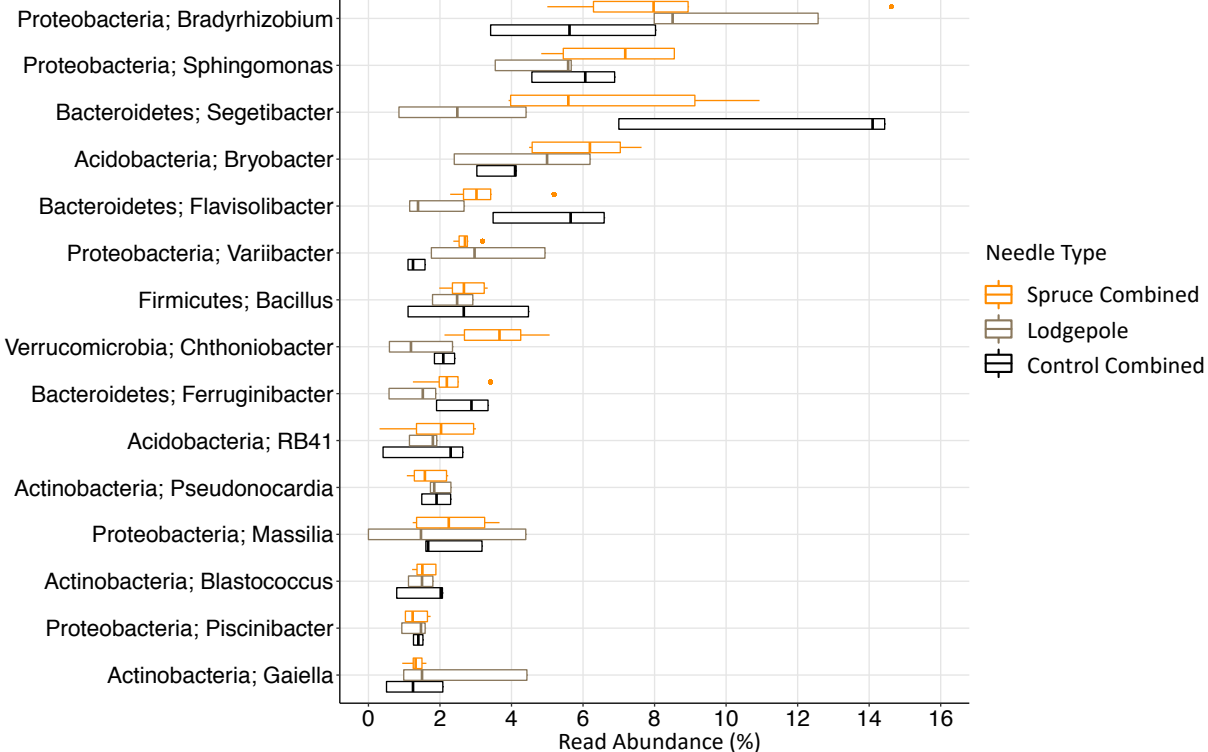

Supplement: Supplemental Information 4 [file peerj-08-9538-s004.pdf]

## Phylum

AUG 2017

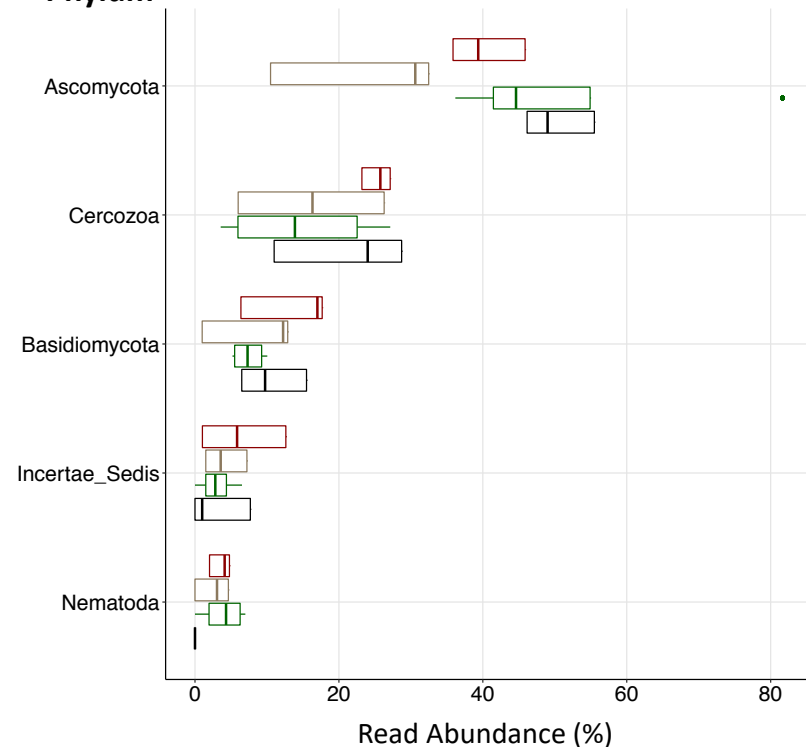

## Phylum

JUL 2018

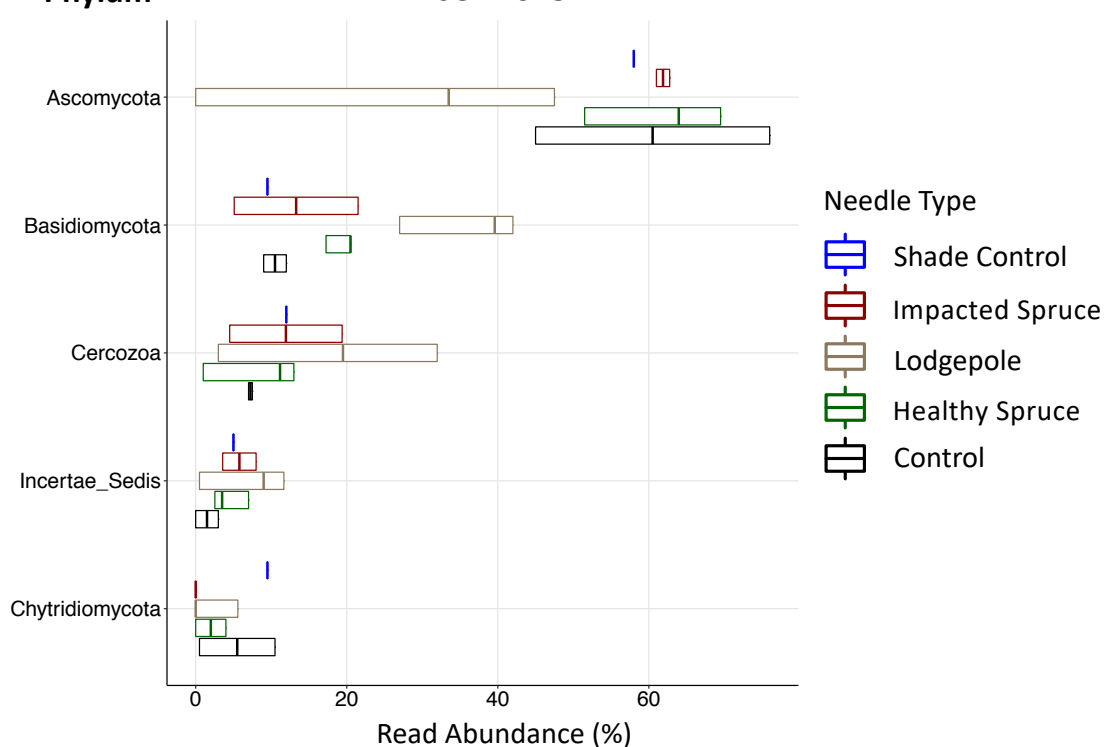

Supplement: Supplemental Information 5 [file peerj-08-9538-s005.pdf]

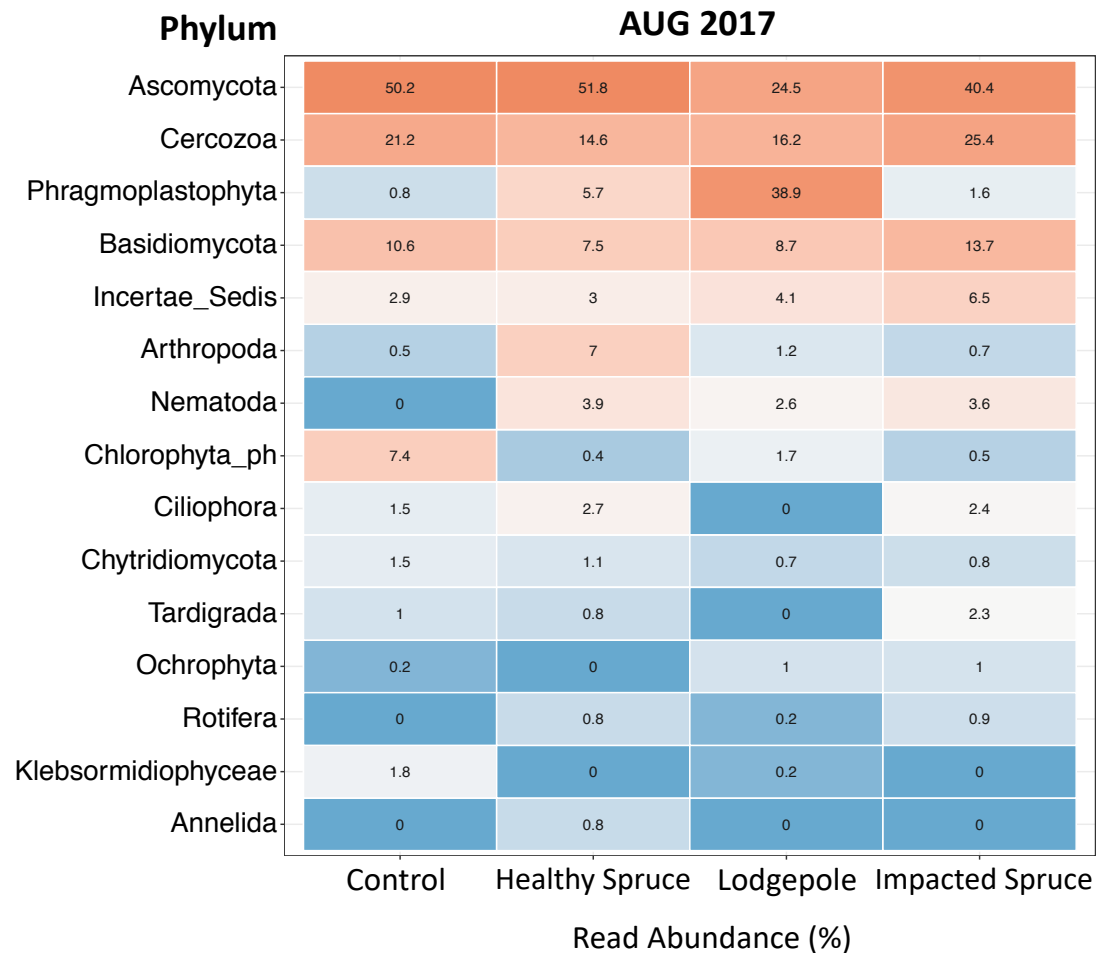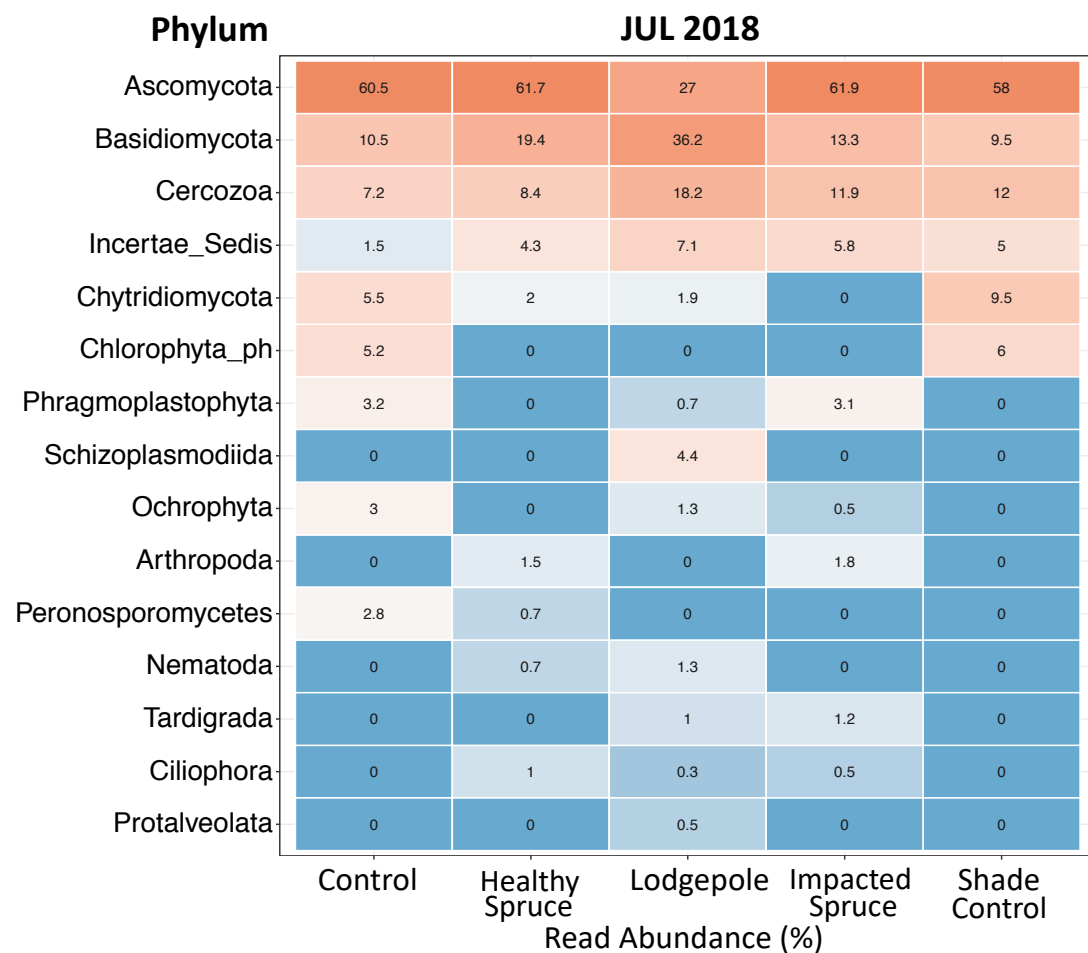

Supplement: Supplemental Information 6 — Samples were not combined for 18S due to more distinct differences between the impacted and healthy spruce. [file peerj-08-9538-s006.pdf]
