# Supplementary material for: A comparison of lodgepole and spruce needle chemistry impacts on terrestrial biogeochemical processes during isolated decomposition
Source: PeerJ. 2020 Jul 16;8:e9538. doi: 10.7717/peerj.9538 (PMC7369028; doi:10.7717/peerj.9538)
Supplement: Supplemental Information 7 [file peerj-08-9538-s007.docx]

| 2016 Initial Needle Litter | | | | |
| --- | --- | --- | --- | --- |
|  | **Healthy Spruce** | **Impacted Spruce** | **Lodgepole** |  |
| Carbon (%) | 45.9 (±0.2) | 51.8 (±0.1) | 53.7 (±0.1) |  |
| Nitrogen (%) | 0.7 (±0) | 0.8 (±0) | 0.9 (±0) |  |
| C:N | 62.7 (±1.7) | 68.2 (±0.2) | 61.6 (±1.1) |  |
| 2018 Decomposed Needle Litter | | | | |
|  | **Healthy Spruce** | **Impacted Spruce** | **Lodgepole** |  |
| Carbon (%) | 38.9 (±15.3) | 48.6 (±0.6) | 51.9 (±0.7) |  |
| Nitrogen (%) | 0.9 (±0.3) | 0.9 (±0) | 0.7 (±0.1) |  |
| C:N | 45.1 (±2.6) | 52.2 (±1.1) | 72.4 (±15.4) |  |

Results are based on an air-dried basis.

Averages plus or minus standard deviation in parenthesis (n=3).
