# Supplementary material for: A comparison of lodgepole and spruce needle chemistry impacts on terrestrial biogeochemical processes during isolated decomposition
Source: PeerJ. 2020 Jul 16;8:e9538. doi: 10.7717/peerj.9538 (PMC7369028; doi:10.7717/peerj.9538)
Supplement: Supplemental Information 8 — Peak area integration values at known functional groups of interest. [file peerj-08-9538-s008.docx]

| 2016 Initial Needle Litter | | | | | | |  |
| --- | --- | --- | --- | --- | --- | --- | --- |
|  | **Healthy Spruce** | | **Impacted Spruce** | | **Lodgepole** | | |
| Ether Linkages (1150 cm^-1^) | 50 (±2) | | 50 (±2) | | 64 (±2) | | |
| Aromatics (1510 cm^-1^) | 33 (±1) | | 30 (±2) | | 19 (±1) | | |
| Amides (1600 cm^-1^) | 193 (±4) | | 182 (±3) | | 180 (±6) | | |
| Carbonyl (1720 cm^-1^) | 38 (±1) | | 44 (±1) | | 72 (±6) | | |
| Cellulose:Lignin | 1 (±0) | | 2 (±0) | | 3 (±0) | | |
|  |  |  |  |  |  |  | |
| 2018 Decomposed Needle Litter | | | | | | |  |
|  | **Healthy Spruce** | | **Impacted Spruce** | | **Lodgepole** | | |
| Ether Linkages (1150 cm^-1^) | 43 (±11) | | 58 (±11) | | 76 (±6) | | |
| Aromatics (1510 cm^-1^) | 16 (±14) | | 21 (±2) | | 13 (±3) | | |
| Amides (1600 cm^-1^) | 171 (±67) | | 193 (±6) | | 143 (±4) | | |
| Carbonyl (1720 cm^-1^) | 23 (±15) | | 40 (±6) | | 84 (±9) | | |
| Cellulose:Lignin | 4 (±3) | | 3 (±1) | | 6 (±1) | | |
|  |  | |  | |  | | |

Results are based on an air-dried basis.

Averages plus or minus standard deviation in parenthesis (n=3).
